# Guillain-Barre Syndrome as an Atypical Early Presentation of Adult-Onset Still's Disease: A Case Report

**DOI:** 10.7759/cureus.62162

**Published:** 2024-06-11

**Authors:** Jamila Al Kaakour, Chirine El-Kojok, Sara El Mustapha, Jean Claude Kheirallah

**Affiliations:** 1 Internal Medicine, Lebanese University Faculty of Medicine, Beirut, LBN; 2 Infectious Diseases, Lebanese University Faculty of Medicine, Beirut, LBN; 3 Internal Medicine and Infectious Diseases, Lebanese Hospital Geitaoui, Beirut, LBN

**Keywords:** adult-onset still's disease, lung involvement, steroids, neurological involvement, guillain-barre syndrome

## Abstract

Adult-onset Still's disease (AOSD) is a rare auto-inflammatory disorder with unknown pathophysiology. Although having a heterogeneous clinical spectrum, the major features of AOSD include fever, rash, and arthritis or arthralgia. Neurological involvement is rare in AOSD with aseptic meningitis being the most common presentation. Guillain-Barre syndrome (GBS) has never been reported as an early presentation of AOSD. Herein, we describe the case of a patient presenting with GBS and fever of unknown origin who was soon diagnosed with AOSD and improved with corticosteroid therapy.

## Introduction

Adult-onset Still's disease (AOSD) is a very uncommon systemic inflammatory disease characterized by high-grade fever, articular involvement, maculo-papular rash, hepatosplenomegaly, and lymphadenopathy [1]. The diagnosis of AOSD is, in part, a diagnosis of exclusion that can be made upon the presence of characteristic clinical and laboratory features in the absence of another condition with similar symptoms. A number of non-specific laboratory findings are characteristic of AOSD including elevation in acute phase reactants including ferritin, with neutrophilic leukocytosis. Anemia and elevated hepatic transaminases are also seen [2]. The elevation in serum ferritin can be striking and is commonly seen at levels that are above those in other disorders and is present in as much as 70% of patients [3]. Particularly, the percentage of glycosylated ferritin in AOSD tends to be lower than in other rheumatic diseases (<20%) [4]. Neurological involvement has been described in 7-12 % of cases with AOSD [5,6] with aseptic meningitis being the most common presentation [7].

We describe the case of a patient presenting with Guillain-Barre syndrome (GBS) treated with plasmapheresis but with persistent fever during hospitalization and finally diagnosed with AOSD after other infectious, oncologic, and autoimmune diseases were excluded.

## Case presentation

A 32-year-old female patient, non-smoker, previously healthy, presented to the ER department for one-week history of sore throat, fever, arthralgia, and myalgia followed by a non-pruritic papular rash over her extremities and worsening proximal muscle weakness with difficulty in walking.

On physical examination, the patient was alert and oriented, she had a temperature of 39° C with stable blood pressure. The respiratory exam was normal and her abdomen was soft. She had a pink-colored papular rash over her extremities. Regarding neurological exam, she had symmetrical cranial nerve exam and normal positional sense, but with 2-3/ 5 motor power in her proximal muscles and 4/5 distal motor power in her upper and lower limbs. She also had depressed patellar tendon reflexes with normal Achilles tendon reflexes.

Regarding her labs, she had a hemoglobin of 14.5 mg/dl and neutrophilic leukocytosis (white count: 13000/mm³, neutrophils: 88%), with a CRP of 250 mg/L. Electrolytes, creatinine, urine analysis, CPK, PT, PTT, INR, and bilirubin were all normal. She had disturbance in liver enzymes with ALP: 344 U/L, GGT: 306 U/L, SGOT: 281 U/L, and SGPT: 378 U/L. Urine and blood cultures were taken. Chest X-ray, CT brain, and abdominal US were all normal. The next day, she had worsening in proximal muscles motor power with 2/5 motor power in both upper and lower extremities and difficulty taking deep breaths with continuous quotidian fever. Lumbar puncture was then done with the following findings (normal OP, glucose: 64 mg/dl, protein: 60 mg/dl, no WBCs or RBCs) but EMG turned out to be negative. The patient was diagnosed with GBS and planned for plasmapheresis (immunoglobulin therapy not available). To rule out the infectious etiology, Brucella, Salmonella, Chlamydia, EBV, CMV, Mycoplasma, coxsackies, Hepatitis C serologies, Hepatitis B antigen levels, urine, and blood culture were drawn and were all negative.

**Figure 1:**
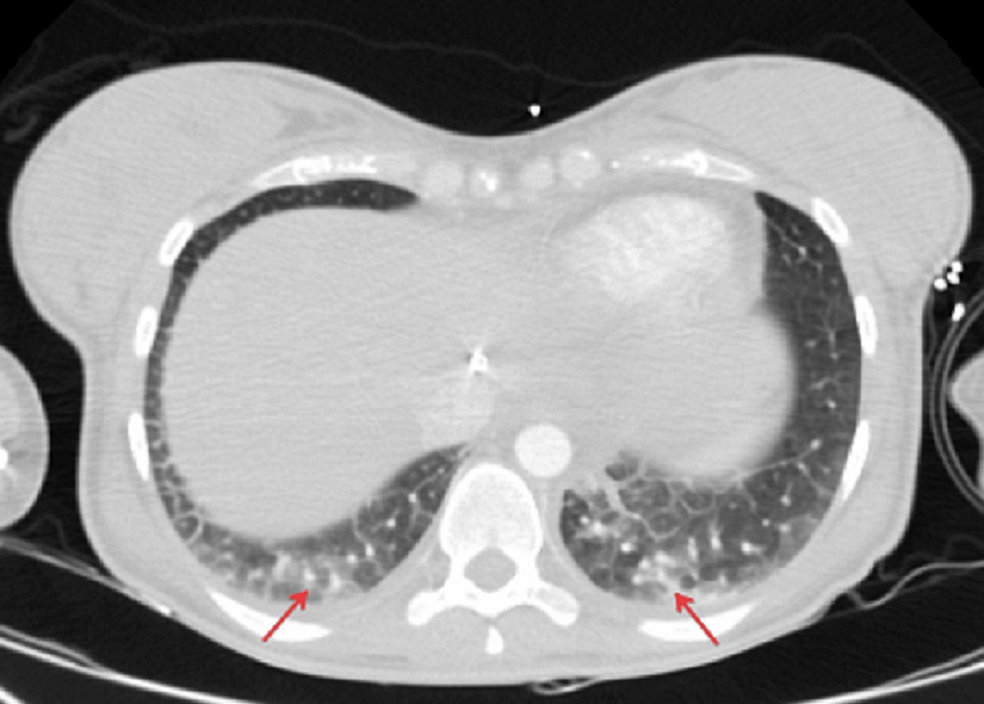
Axial CT image of the thorax with lung algorithm demonstrates multiple poorly defined centrilobular nodules in the lower lobes, mainly on the left side with a tree-in-bud appearance (red arrows).

During the first plasmapheresis session, she had a decreased level of consciousness and hypotension that was responsive to fluids. Urgent CT brain, chest, abdomen, and pelvis were done; CT chest showed small bilateral pleural effusion and multiple poorly defined centrilobular nodules in the lower lobes, mainly on the left side with a tree-in-bud appearance compatible with infectious process or cardiac origin (Figure 1). Transthoracic echocardiography was normal. Thus, the patient was diagnosed with pneumonia, despite the absence of respiratory symptoms and was started on vancomycin and piperacillin/ tazobactam in addition to azithromycin.

At this time, the ferritin level came back to 15000 ng/dl. From the differentials of this striking ferritin level, AOSD was one of the most possible diagnoses so the glycosylated ferritin level was requested.

In the following days, her motor power was totally improving after five sessions of plasmapheresis; however, she still had fever, rash, elevated liver enzymes, neutrophilic leukocytosis (reaching 25000 / mm³), and elevated CRP despite negative cultures.

Macrophage activating syndrome was feared but with normal fibrinogen, normal platelets (200,000), normal triglycerides (125 mg/dl), and absence of hepatosplenomegaly, it was ruled out. Hemolytic workup, including haptoglobin (2.39 SI unit), reticulocyte count (2%), peripheral smear with 1% myelocytes, bilirubin, and Coombs test, was normal. 

After excluding the infectious etiology of her fever, which was even non-responsive to non-steroidal anti-inflammatory drugs, she was started on IV glucocorticoids (methyl prednisone 80mg/per day), with a remarkable decrease in fever episodes per day and later on colchicine was added and antibiotics were stopped. CT chest control showed resolution of previously described findings. Autoimmune, vasculitis, and hematological malignancy causes were investigated with no positive findings: P-ANCA: 0.5 AU/ml, C-ANCA: 2.3 AU/ml, C3: 2.4 g/L, C4: 0.6 g/L, negative ANA, ds-DNA, RF and anti- CCP, normal serum protein electrophoresis, and peripheral immuno-chemistry. Finally, glycosylated ferritin turned out to be 9% (1350 ng/dl).

Here a diagnosis of AOSD was made, and the patient was maintained on IV steroids with no more fever. Then she was discharged home on PO steroids (prednisone 40 mg per day) and colchicine.

After one month of home steroids, there was complete bio-chemical remission as shown in the following labs: hemoglobin: 13 mg/dl, WC: 8000/mm³, CRP: 2 mg/L, ferritin: 237 ng/ml and normalization of liver enzymes and steroids was tapered.

## Discussion

AOSD is a rare systemic inflammatory disorder with a heterogeneous clinical spectrum. The etiology of AOSD is unknown; both genetic factors and a variety of infectious triggers have been suggested. A retrospective French study estimated the annual incidence of AOSD to be 0.16 cases per 100,000 people, with an equal distribution between both sexes [8].

The major clinical features of AOSD include fever, rash, and arthralgia each occurring in about 71-95% of patients [1]. The fever of AOSD is usually quotidian and it usually precedes other manifestations [9].

In the published literature, neurological involvement has been described in 7-12 % of cases with AOSD [5,6]. They mainly included seizures, aseptic meningitis or encephalitis, brainstem hemorrhage, cranial nerve paralysis, pyramidal tract signs, hemiplegia, reversible posterior leukoencephalopathy syndrome, ischemic stroke, sensorineural deafness, and symmetrical peripheral nerve abnormalities [1,3,10-12]. Neurological damage is usually thought to be seen in the late stage of the disease. Our patient complained of muscle weakness during the first days of the disease. To our knowledge, this is the first reported case of GBS described as an initial presentation of AOSD.

GBS is an acute immune-mediated polyneuropathy characterized by progressive and symmetrical muscle weakness with absent or depressed deep tendon reflexes. Patients may also have sensory symptoms and dysautonomia. Miller Fisher, a variant of GBS, has been described in a patient five years after being diagnosed with AOSD [13]. Classical GBS has been reported in a patient two years after AOSD diagnosis [14].

In a previous retrospective Japanese study, a total of 187 AOSD cases were reviewed. The prevalence of neurological involvement was 7.5% with aseptic meningitis being the most common presentation (64.3%). Other neurological manifestations included cranial nerve palsy, encephalitis, and cerebral infarction. Neurological symptoms were the initial manifestations of AOSD in 28.6% of cases [7].

Parenchymal lung involvement is present in less than 5% of AOSD cases as described in the case series of Gerfaud-Valentin et al. [15] and ranges from nonspecific reticular interstitial opacities to life-threatening conditions, such as acute respiratory distress syndrome.

The most frequent respiratory symptoms in patients with non-acute respiratory distress syndrome were cough, dyspnea, and chest pain, while the main high-resolution CT patterns were non-specific interstitial pneumonia, organizing pneumonia, and unclassified interstitial lung disease. This may explain the pneumonia found in the high-resolution CT scan in our patient and the non-responsiveness to IV antibiotics.

## Conclusions

The diagnosis of AOSD needs an extensive workup to rule out infections, malignancy, systemic autoimmune diseases, vasculitis, and drug reactions. Neurological involvement can complicate the picture further; however, clinicians should be aware of those rare manifestations of AOSD. Whether GBS and AOSD share a common trigger or are two different entities remains questionable, and this should be targeted in the upcoming research studies.
